# Evaluation of ebselen supplementation on cryopreservation medium in human semen

**Published:** 2014-04

**Authors:** Zohreh Khodayari Naeini, Hassan Hassani Bafrani, Hossein Nikzad

**Affiliations:** 1 *Kashan Anatomical Sciences Research Center, Kashan University of Medical Sciences, Kashan, Iran.*; 2 *Gametogenesis Research Center, Kashan University of Medical Sciences, Kashan, Iran.*

**Keywords:** *Cryopreservation*, *Ebselen*, *DNA fragmentation*, *Human sperm*

## Abstract

**Background:** An effect of cryopreservation on human sperm is sublethal cryodamage, in which cell viability post-thaw is lost more rapidly at later times than in fresh cells.

**Objective:** This study examined whether the addition of an antioxidant to cryopreservation medium could improve the post-thaw parameters and evaluation of sperm chromatin quality of cryopreserved human spermatozoa from men with normal semen parameters.

**Materials and Methods: **Semen samples (n=35) were collected by masturbation and assessed following WHO standards. Individual samples were classified as two portions. One portion (n=10) was for elucidate the concentration of ebselen.Then the samples(n=25) were divided in to 5groups.The first aliquot remained fresh.The second aliquots was mixed with cryopreservation medium.The third aliquots were mixed with cryopreservation medium containing solvent of ebselen.The forth and fifth aliquots were mixed with cryopreservation medium containing 1.25 and 2.5 µm of ebselen.Samples were frozen and thawed samples were assessed for sperm parameters.Three-way ANOVA Multivariate measures were used to assess. According to this assesment the differences are observed in existent groups in post-thaw count, motility index, vitality staining, and morphology and DNA fragmentation.

**Results: **After freezing the media containing of ebselen, DNA fragmentation is significantly different in comparison with control group. ebselen with 1.25 µm dose was significantly associated with post-thaw DNA fragmentation (p=0.047). Similarly ebselen with 2.5 µm dose was significantly associated with post-thaw DNA fragmentation (p=0.038). But other parameters were not altered.

**Conclusion:** These results suggest that the addition of ebselen to cryopreservation medium doesnot improve post-thaw parameters and DNA fragmentation of sperm.

## Introduction

“Cryopreservation of human spermatozoa has evolved as an important area in assisted reproductive technology programs. cryopreservation of human semen has been widely used as a vital method for the fertility preservation of male patients before they undergo chemotherapy, radiotherapy, and/or surgery that may lead to testicular failure or ejaculatory dysfunction (1, 2). Freezing of sperm before initiation of treatment provides patients with ‘‘fertility insurance’’ and may allow them to father their own children through the use of intrauterine insemination, especially by cryopreserved donor semen, conventional in vitro fertilization or intracytoplasmic sperm injection. 

Recently, sperm cryopreservation has become an important component of assisted reproduction. Sperm preparation for cryopreservation involves the removal of seminal plasma and consequently the predominant source of antioxidant protection. Freeze-thawing of equine spermatozoa is also associated with an increase in of reactive oxygen species (ROS) generation (3). Cryopreservation can induce an increase rate of lipid peroxidation in the sperm plasma membrane causing an overall increase in the concentration of oxygen radical in the plasma (4). Cooling and thawing of spermatozoa cause an increase in the generation of superoxide radicals. Increase in lipid peroxidation levels have been observed in frozen/thawed spermatozoa and appears to be associated with a reduction in sperm membrane fluidity as detected by spin labeling studies (5). 

Despite several efforts to improve the sperm cryosurvival, decrease in motility of 25-75%, sperm DNA fragmentation, and reduction in sperm function are commonly observed after thawing (6, 7). Loss of sperm function and DNA fragmentation during cryopreservation has been linked to the production of ROS during the freeze-thaw process (5). High polyunsaturated fatty acid content in the plasma membrane and a limited free radical scavenging system in the cytoplasm make spermatozoa highly susceptible to free radical assault. Spermatozoa are protected by various antioxidants and antioxidant enzymes in the seminal plasma or in spermatozoa itself to prevent oxidative damage (8). An antioxidant that reduces oxidative stress and improves sperm motility could be useful in the management of male infertility (9). Antioxidants are the agents, which break the oxidative chain reaction, thereby, reduce the oxidative stress (10, 11). 

Supplementation of antioxidant molecules, antioxidant enzymes, or both to freezing media has been tried previously to improve the post-thaw survival and DNA integrity of spermatozoa (12, 13). Ebselen, 2-phenyl-1,2-benzisoselenazol-3 (2H)-one, is a cyclic OS, which exhibits interesting therapeutic potential against a number of disease states involving oxidative stress, such as neurological disorders, acute pancreatitis, noise induced hearing loss and cardiotoxicity, due to antioxidant, cytoprotective, neuroprotective and anti-inflammatory activities (-). Ebselen is a poor radical scavenger, if at all, but it is an effective scavenger of organic hydroperoxides, in particular, of lipid hydroperoxides. 

Thus, the particular interest in this drug is that ebselen mimics glutathione peroxidase (GPx) activities in particular that of phospholipid hydroperoxide GPx (19). The aim of this study therefore is to determine whether the supplementation of cryopreservation medium with ebselen can improve post thaw integrity of cryopreserved human spermatozoa.

## Materials and methods

Semen samples were obtained by masturbation and collected into sterile containers after 3-5 days of abstinence from sexual activity. Semen inclusion criteria were as follows: concentration >60×10^6 ^/ml; motility>50%; forward motility >20%; total motile sperm count> 40×10^6^ and strict morphology score>10%. After 10-15 minutes of liquefaction at 37^o^C with 5% CO_2_ in air, semen samples were examined for volume, sperm concentration, morphology and motility according to the World Health Organization (WHO) guidelines.

Written approval for this study was granted by the Ethics Committee in Kashan University of Medical Sciences. This study utilized semen remaining after routine semen analysis at the Kashan fertility and infertility center in the Shahid Beheshti hospital. Semen samples were collected by masturbation into sterile containers. Patient consent to use surplus semen was obtained prior to use.

**Semen analysis**

Semen analysis was performed according to the World Health Organization (WHO) guidelines using light microscopy (20). Concentration was determined by using a haemocytometer (Improved Neubauer, Weber, England), motility was determined by using four categories of movement; fast progressive, slow progressive, non-progressive and immotile and morphology by using Diff-Quik (Fronine, Sydney, Australia) stain. Vitality was assessed using trypan blue stain.

**Preparation of stock solutions**

Ebselen was purchased from Sigma (St Louis, MO, USA). Ebselen was initially solved in dimethyl sulphoxide (DMSO) (Sigma-Aldrich, Missouri, USA) to yield a 10 mM stock solution. This solution was frozen in aliquots and stored at -20^o^C until the time of using. A 1 mM working solution was prepared at the time of use with Quinn's Advantage® Medium with HEPES (QAMH) (SAGE In-Vitro Fertilization Inc, Connecticut, USA).

**Study design**

In this experimental study, To elucidate the concentration of ebselen at which maximum sperm cryosurvival is offered, liquefied ejaculates from normozoospermic semen samples (n=10) were mixed with an equal volume of washing media supplemented which contains various concentration(0.6, 1.25, 2.5, 5, 10 µM) of ebselen solution. Maximal in the 1.25 and 2.5-µM groups compared with the other concentration groups. The motility and count declined with an increase in ebselen concentration >2.5 µM (Table I). Then semen sample (n=25) was equally divided into 5 groups. The forth and fifth groups received the antioxidant ebselen (1.25 and 2.5µM). Each semen sample was first cryopreserved and then thawed. After freeze-thaw treatment, spermatozoa were examined for their general quality and DNA damage: (Sperm Chromatin Dispersion Test).

**Cryopreservation and thawing of spermatozoa**

All samples were loaded into de-identified 0.5 ml Cassou straws (IMV Technologies, L’Aigle, France) and sealed with Stopping Powder (IMV Technologies). All straws were frozen using Rapid Freezing method. Rapid Freezing was first proposed by Sherman (21). This technique requires direct contact between the straws and the nitrogen vapours for 8-10 min and immersion in liquid nitrogen at -196^o^C. The sample is initially mixed in drop wise manner with equal volume of cold cryoprotectant; the mixture is loaded into the straws and left to incubate at 4^o^C for 10 minutes. After this stage, the straws are immersed in liquid nitrogen. During cooling it is preferable to place the straws in horizontal position to minimize the heat difference between the two ends. Samples were removed from storage in batches and thawed at room temperature.

**Assessment of sperm DNA fragmentation**

**Sperm Chromatin Dispersion** (**SCD) Test**

Aliquots of 0.2 mL of raw semen and of the different Isolate gradient fractions in mHTF medium were either analyzed directly or frozen in liquid nitrogen prior to analysis. Samples were thawed at room temperature and diluted in mHTF medium to obtain sperm concentrations that ranged between 5 and 10 million/mL. The suspensions were mixed with 1% low-melting point aqueous agarose (to obtain a 0.7% final agarose concentration) at 37^o^C. Aliquots of 50 mL of the mixture were pipetted on to a glass slide precoated with 0.65% standard agarose dried at 80^o^C, covered with a coverslip (24 by 60 mm), and left to solidify at 4^o^C for 4 min. As in the halo test or the comet assay, the agarose matrix allows for work with unfixed sperm on a slide in a suspension like environment. Coverslips were carefully removed, and slides were immediately immersed horizontally in a tray with freshly prepared acid denaturation solution (0.08 NHCl) for 7 minutes at 22^o^C in the dark to generate restricted single-stranded DNA (ssDNA) motifs from DNA breaks. 

The denaturation was then stopped, and proteins were removed by a transfer of the slides to a tray with neutralizing and lysing solution 1 (0.4 M Tris, 0.8 M DTT, 1% SDS, and 50mM EDTA, pH=7.5) for 10 minutes at room temperature, which was followed by incubation in neutralizing and lysing solution 2 (0.4 M Tris, 2 M NaCl, and 1% SDS, pH=7.5) for 5 minutes at room temperature. Slides were thoroughly washed in Trisborate-EDTA buffer (0.09 M Tris-borate and 0.002 M EDTA, pH= 7.5) for 2 min, dehydrated in sequential 70%, 90%, and 100% ethanol baths (2 minutes each), and air dried. 

Cells were stained with the Diff-Quik reagent (Baxter Healthcare Corporation Inc,McGaw, Ill) for bright field microscopy. Sperm were evaluated manually on each slide for halo size and dispersion pattern as described by Ferna´ndez *et al*; 1) nuclei with large DNA dispersion halos, 2) nuclei with medium sized halos (Figure 2), 3) nuclei with small sized halos, and 4) nuclei with no halo (22). The nuclei with large to small size halo were considered sperm with nonfragmented DNA, whereas nuclei with no halo were considered sperm with fragmented DNA.

**Statistical analysis**

Three-way ANOVA statistical analysis method was used to assess differences between fresh and freeze-thawed sperm within each parameter. Values are therefore expressed as medium±SEM range. Statistical differences were considered to be significant if p≤0.05. All analyses were performed using the SPSS 16 statistical software.

## Results

Semen parameters of the samples used in this study are presented in table I and II. Individual samples were classified as two portions. One portion (n=10) was for elucidate the concentration of ebselen. In this part, the samples were divided in to 8 groups. The results are shown in table I. According to it, the sperm count and total motility in groups 1.25 and 2.5 µM of ebselen in comparison to other groups is significantly different.

Total sperm concentration and motile sperm concentration normality were significantly higher (p<0.001), as compared with other doses of ebselen. After determining the suitable dose in the previous stage (1.25 and 2.5 µM), the samples (n=25) were divided in to 5 groups (Table II). There was a significant main effect before freezing group (group one) and after freezing groups (F [7, 62]=4.790, p=0.0001), that post-thaw count was significantly decreased over control. However there was no improvement in the group with medium freezing treated with doses of ebselen (groups four and five). 

**Figure 1:**
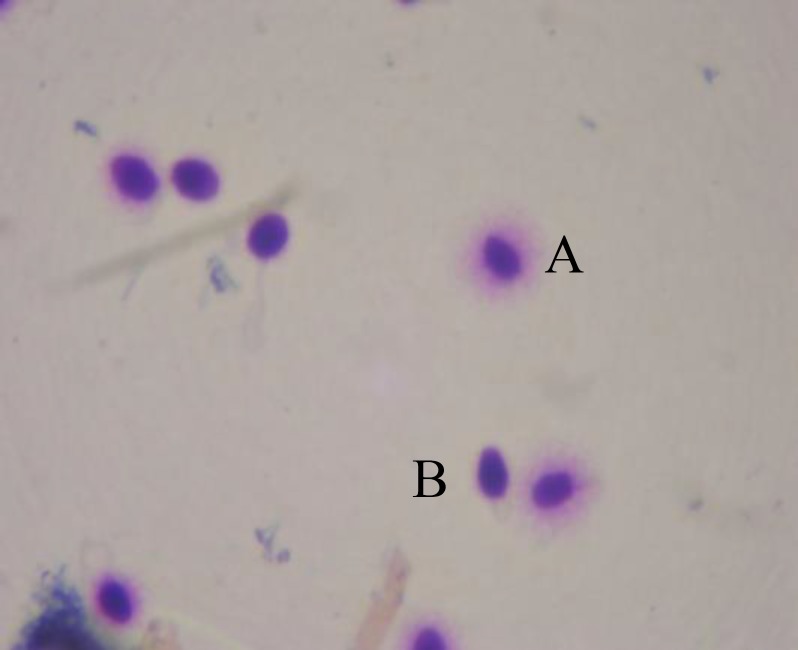
Sperm chromatin dispersion test in fresh group that showes nuclei with large DNA dispersion halos (A) and nuclei with no halo (B). (magnification of ×1000)

**Figure 2:**
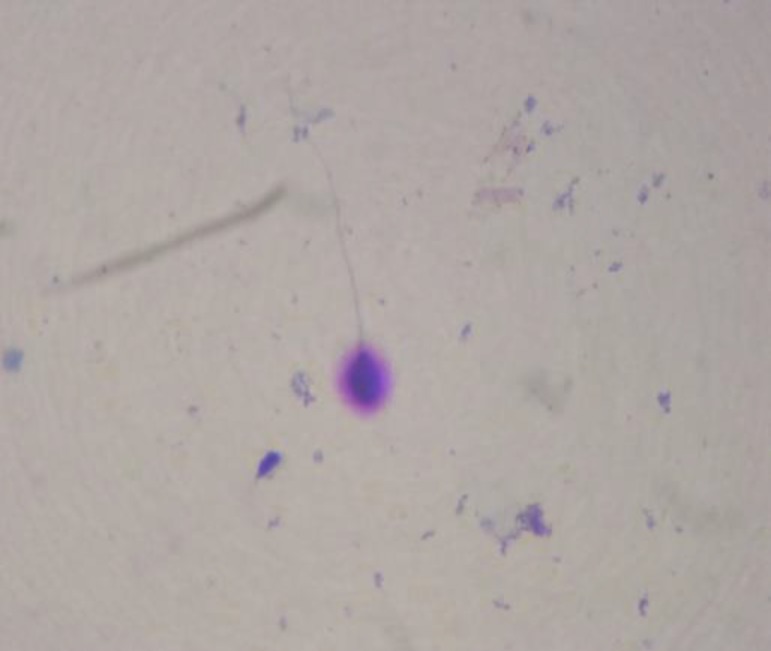
The medium halo of sperm in after freezing group. (magnification of ×1000)

There was no significant differences in motility between before (group one) and after freezing groups (F [7, 62]=0.393). However, there was an improvement in the group with medium freezing treated with 1.25 µm of ebselen (group four). Sperm chromatin dispersion is significantly lower in after freezing groups (F [5,44]=6.565, p=0.006) (Figure 2)compared with before freezing group (group one) (Figure 1). In after freezing groups it is observed that significant decrease in the group with medium freezing treated with 1.25 µm of ebselen (group four) (p=0.047) and in the group with medium freezing treated with 2.5 µm of ebselen (group five) (p=0.038) (Table II).

**Table I:** Three-Way ANOVA test of Semen count and motility of the samples to elucidate the concentration of ebselen

**Groups**			**Count (million)**	**Total motility (%)**
Control			63 ± 0.77	86 ± 0.41
Solvent			39 ± 0.90	76 ± 0.51
Ebselen (MM)				
	0.6		28 ± 0.90	70 ± 0.45
	1.25		65 ± 0.75^a^^, ^b	87 ± 0.60^a^^, ^b
	2.5		64 ± 0.90^a^^, ^b	86 ± 0.45^a^^, ^b
	5		31 ± 0.87	65 ± 0.45

Values are the mean±SEM

a p≤0.001 compared with 0.6 and 5µm doses of ebselen.

b p≤0.01 compared with solvent group.

**Table II:** Three-Way ANOVA test of Semen parameters of the samples used in this study

**Group**	**Fresh (1)**	**Freeze (2)**	**Freeze+ medium freezing treated with solvent (3)**	**Freeze+ medium freezing treated with 1.25µm of ebselen (4)**	**Freeze+medium freezing treated with 2.5µm of ebselen (5)**
Count (million)	39.44 ± 27.86	12.56 ± 0.36 a	8.39 ± 0.32 a	9.33 ± 0.03 a	9.56 ± 0.03 a
Viability (%)	81.89 ± 13.07	83.67 ± 0.34	84.44 ± 0.42	84.44 ± 0.42	86.5 ± 0.33
Total motility (%)	94.00 ± 4.30	66.89 ± 0.59	62.33 ± 0.57	66.33 ± 0.62	66.75 ± 0.63
Morphology (% normal)	18.78 ± 2.99	24.11 ± 0.35	24.89 ± 0.35	25.67 ± 0.35	24 ± 0.38
Sperm chromatin dispersion*	23.44 ± 6.064	38.89 ± 0.46 c	43.00 ± 0.51^c^	53.44 ± 0.36^a^^b^	54.63 ± 0.40^a^^, ^^b^

Values is the mean±SEM.

* % fragmented

a p<0.0001 compared with fresh semen samples in that line.

b p<0.05 compared with freeze samples in that line.

c p<0.05 compared with fresh samples in that line

## Discussion

Although the cryopreservation of human semen is an important technique routinely employed in the clinical management of male infertility, the relevant cryodamage remains a great challenge (23). The aim of the present study is to determine whether the addition of an antioxidant to the semen cryopreservation medium can improve the post-thaw integrity of cryopreserved human spermatozoa. It was found that ebselen supplementation of semen cryopreservation medium at a concentration of 1.25 µm and 2.5 µm does not improve post-thaw count, motility, viability and morphology of sperm. We have shown that adding the ebselen antioxidant to cryopreservation medium is detrimental to sperm chromatin.

Cryopreservation can result in increased lipid peroxidation in human spermatozoa and has been shown to reduce antioxidant defenses in bovine spermatozoa (24). Because excessive ROS was generated during the cryopreservation process (5, 25). In recent years, a variety of cryoprotective media, mostly supplemented with antioxidants, have been designed in an attempt to overcome the cellular damage caused by cryopreservation (26, 27). The newer cryoprotective media have been shown to be useful for inhibition of ROS generation in some experiments (28). We evaluated further the effect of the antioxidants ebselen for human spermatozoa. 

Ebselen is effective scavenger of organic hydroperoxides, in particular, of lipid hydroperoxides. Thus, the particular interest in this drug is that (GPx) activities in particular that of phospholipid hydroperoxide GPx (29). In the present study, we evaluated the potential benefits of ebselen for human sperm cryopreservation. The result showed that 1.25 µm and 2.5 µm ebselen supplementation does not improve the motility, morphology, viability and DNA damage. Chromatin condensation is also vital for spermatozoa because of the fact that spermiogenesis results in the discarding of cytoplasm, causing cessation of transcription and leaving the spermatozoa incapable of undertaking DNA repair. Hence, the assessment of sperm DNA damage related to freeze/ thawing is very important (30). 

The present study demonstrated that cryopreservation also leads to DNA damage. Collectively, these results strongly indicate that the cryopreservation process indeed led to a variety of structural and functional injury to human spermatozoa. Possible mechanisms for the cryodamage to human spermatozoa are thought to be multifactorial, but the excessive ROS production during freezing and thawing has been previously demonstrated to be a significant contributing factor (5). ROS can also damage the DNA in the sperm nucleus (4). In contrast, Jiang *et al* did not report any adverse effect of cryopreservation on sperm DNA, and Donnelly et al reported that only spermatozoa from infertile males demonstrated a significant increase in DNA fragmentation following cryopreservation (31).

In the present study, the addition of ebselen to the cryopreservation medium did not affect post-thaw vitality or DNA fragmentation. Donnelly et al showed that addition of glutathione and hypotaurine, either singly or in combination, to sperm preparation medium had no significant effect on sperm progressive motility or baseline DNA integrity. Despite this, sperm were still afforded significant protection against H_2_O_2_ induced damage and ROS generation. Cryopreservation protocols and extender formulations vary among laboratories and among species and may account for the differences observed. Donnelly *et al* also showed progressive motility, average path velocity, curvilinear velocity, straight-line velocity, and linearity were decreased significantly, by supplementation in vitro with ascorbate and α-ocopherol, with the greatest inhibition observed with the highest concentrations of antioxidants (32). 

LI *et al* showed appropriate ascorbate or catalase supplementation of cryoprotective medium restrains ROS levels and the resultant cryodamage (30). Branco *et al* showed the use of ascorbic acid and resveratrol did not induce any change in post-thawing sperm concentration or morphology, but both antioxidant were not able to prevent motility decrease induced by the cryopreservation process (33). Taylor *et al* showed vitamin E supplementation of semen cryopreservation medium significantly improved post-thaw motility, but not vitality or the degree of DNA fragmentation (34). “Garcez showed the cryopreservation process was not able to change sperm concentration or morphology. However, a decrease in sperm motility was observed in both the fertile and infertile men. The addition of resveratrol was not able to prevent this effect” (35). 

Iwanier and Zachara showed selenium supplementation did not improve the spermatozoa quality characteristics of sperm count, motility and, morphology (36). Several studies have demonstrated that ebselen is an anti-inflammatory and anti-oxidative agent. Contrary to this, studies have also shown a high degree of cellular toxicity associated with ebselen usage, the underlying mechanism of which remains less understood. In research by Azada suggest that ebselen functions through activation of DNA damage response, alterations in histone modifications, activation of checkpoint kinase pathway and derepression of ribonucleotide reductases (DNA repair genes) which to the best of our knowledge is being reported for the first time (37).

There recently has been much debate regarding the potential advantages of antioxidant therapy in improving male fertility (-). However, it is recognized that this may be a double-edged sword, with considerable undesirable effects if a safety threshold dosage is surpassed (40). It also has been stated that although the role of antioxidant in sperm function is fascinating, further research is required before we can be optimistic about a potential role for antioxidants in the treatment of male infertility (41). This emphasize the view that antioxidant therapy may indeed be threshold concentration in exceeded (40).

This study showed that, although supplementing sperm preparation media with ebselen can improve sperm count and motility, but there are no beneficial effect observed on sperm parameters after freezing. In addition, supplementation with ebselen after freezing has a significant detrimental effect on sperm chromatin. Further studies are currently being performed to determine whether there are any antioxidants that have beneficial effect on sperm parameters. Further examinations of the possible interactions between antioxidants and semen parameters are warranted.

## Footenote

***This article extracted from M.Sc. thesis. (Zohreh Khodayari Naeini)***
